# Rapid Resolution of Recalcitrant Headache With Pasireotide in an Adult Patient With Acromegaly

**DOI:** 10.1210/jcemcr/luae142

**Published:** 2024-08-09

**Authors:** Zeinab Dabbous, Zaina Rohani, Abeer Kaled Abdalrubb, Yaman Alkailani, Rosario Pivonello, Tarik Elhadd

**Affiliations:** Endocrine Section, Department of Medicine, Hamad Medical Corporation, PO Box 3050, Doha, Qatar; Endocrine Section, Department of Medicine, Hamad Medical Corporation, PO Box 3050, Doha, Qatar; Endocrine Section, Department of Medicine, Hamad Medical Corporation, PO Box 3050, Doha, Qatar; Radiology Department, Hamad Medical Corporation, PO Box 3050, Doha, Qatar; Dipartimento di Medicina Clinica e Chirurgia, Sezione di Endocrinologia, Università Federico II di Napoli, 80131 Naples, Italy; Endocrine Section, Department of Medicine, Hamad Medical Corporation, PO Box 3050, Doha, Qatar

**Keywords:** acromegaly, headache, pasireotide

## Abstract

Acromegaly is a chronic hormonal disorder caused by excessive GH secretion. In addition to physiological symptoms, it is often accompanied by debilitating headaches. Although effective treatment options exist, achieving complete symptom control and disease management can still be challenging. This case report chronicles the clinical journey of a 38-year-old male diagnosed with acromegaly in 2013. Despite prior interventions, including surgery and treatment with first-generation somatostatin analogues, severe frequent headaches persisted. Following a switch to pasireotide, the patient reported rapid and complete resolution of headaches and normalization of IGF-1 levels within a month of the treatment switch. This report underscores the challenges in acromegaly management and confirms the potential utility of pasireotide for patients suffering from treatment-resistant headache.

## Introduction

Acromegaly is a rare endocrine disorder characterized by a chronic hypersecretion of growth hormone (GH) from a pituitary adenoma (1). GH induces the synthesis of insulin-like growth factor-1 (IGF1), and elevated levels of both cause metabolic dysfunction and somatic growth, which are associated with significant morbidity and mortality (2-4). Symptoms are caused by the mass effect of the pituitary adenoma as well as from systemic effects of the elevated levels of GH and IGF1 (2). In addition to morphological changes, headache is a common sign, occurring in 59% of patients at diagnosis (1).

Injectable somatostatin analogues (somatostatin receptor ligands) are routinely recommended by guidelines for the medical management of acromegaly following surgery (5-7). The somatostatin analogues differ in their binding affinities for the 5 somatostatin receptors. Long-acting, first-generation somatostatin analogues, octreotide long-acting release (LAR), and lanreotide autogel are currently considered the first choice medical treatment following surgery or where surgery is inappropriate (8). Octreotide is highly selective for somatostatin subtype receptor (SST)2 and to a lesser extent to SST5 (8). The second-generation somatostatin analogue, pasireotide (Signifor LAR) has a higher affinity for SST3 and SST5 (which have been shown to be expressed on GH-secreting adenomas) and half the affinity of octreotide for SST2 (8). Pasireotide has been shown to be more effective in achieving biochemical control than octreotide or lanreotide (2, 8-10). However, because pasireotide is associated with a higher incidence of diabetes mellitus, it is generally not prescribed as first-line medical therapy and is used in patients not controlled on first-generation somatostatin analogues, especially when there is a concern with the tumor mass and glucose metabolism is normal (8). Nevertheless, pasireotide was approved by the US Food and Drug Administration for patients with acromegaly who have had an inadequate response to surgery and/or for whom surgery is not an option, and in some Middle Eastern and North African countries as 1 of the treatments for acromegaly patients for whom medical treatment is appropriate. Thus, pasireotide may be potentially used as first-line medical therapy after failed surgery. This is highlighted by recent literature on personalized management of acromegaly where pasireotide is recommended for use in first-line treatment for patients with certain tumor characteristics that predict resistance to first-generation somatostatins (such as magnetic resonance imaging (MRI) T_2_ hyperintensity; sparsely granulated tumor; low or negative SSTR2; high SSTR5 positivity; young age; low aryl hydrocarbon receptor-interacting protein mutations; significant tumor volume; low e-cadherin, and high KI-67) (11-13).

Here, we report a case of a patient with acromegaly with resistant symptoms, most notably intractable headaches despite treatment with octreotide, who was treated with long-acting pasireotide and achieved rapid biochemical control and had complete resolution of the frequent, severe headaches in the first month of treatment.

## Case Presentation

A 38-year-old Jordanian male presented to our clinic in Doha in August 2021. He had been previously diagnosed with acromegaly in Jordan in 2013 following investigation for severe headaches, general weakness, and coarsening of his features. At that time, MRI scanning revealed a pituitary macroadenoma (1.5 × 1.7 × 2.4 cm) and he underwent transsphenoidal surgery. A his 3-month follow-up, MRI showed residual tumor (1.1 × 1 × 1 cm). His IGF1 was also mildly elevated but as the patient claimed to be feeling much better, he did not take any further treatment. In 2015, his headaches and weakness recurred and his IGF1 level was elevated: so he was started on lanreotide, with the dose increased to 120 mg every 4 weeks, but his headaches did not improve and IGF1 remained elevated. He continued on lanreotide until 2017 when he underwent radiation therapy (external beam radiation therapy as 54 Gy/30 fx). After radiation therapy, the patient decided to stop lanreotide by himself as he was hoping he would benefit from the radiation therapy. A pituitary MRI scan in 2018 showed unchanged residual tumor. His IGF1 level was 545 μg/L (71.25 nmol/L)(normal range, 99-239 μg/L [12.94-31.25 nmol/L]). Lanreotide 120 mg every 4 weeks was restarted.

## Diagnostic Assessment

**Figure 1.**
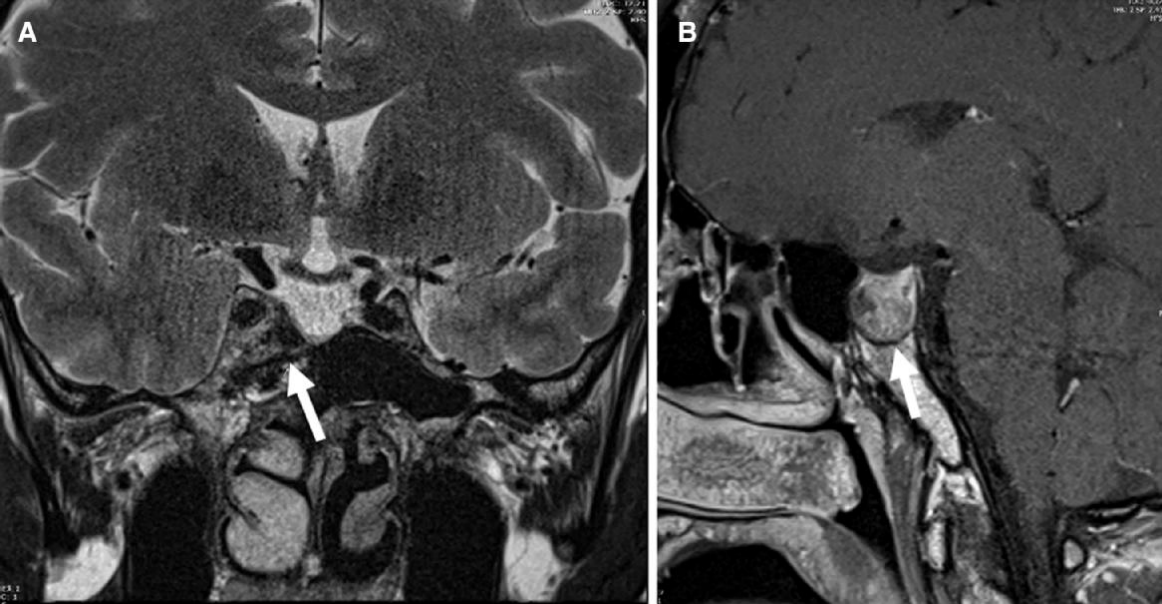
Cross-sectional (A) and sagittal (B) MRI scans showing residual pituitary tumor (December 2021).

In August 2021, he presented to our pituitary clinic in Qatar for treatment of headache and other symptoms associated with acromegaly. His initial biochemical panel results were: IGF1, 89.42 nmol/L (684 μg/L); GH, 6.6 μg/L; testosterone, 7.6 nmol/L (219 ng/dL) (normal range, 10.4-37.4 nmol/L [300-1009 ng/dL]); cortisol level, 283 nmol/L (10.25 mcg/dL)(normal range, 138-689 nmol/L [5-24.9 mcg/dL]); TSH, 0.48 mIU/L (normal range, 0.3-4.2 mIU/L); FT4, 17.5 pmol/L (13.59 ng/L)(normal range 11-23.3 pmol/L (8.55-18.10 ng/L)); prolactin 5.22 μg/L (111 mIU/L)(normal range 4.00-15.18 μg/L [85-323 mIU/L]); and hemoglobin A1c, 5.6%. In December 2021, a pituitary MRI scan showed a residual pituitary adenoma in the right cavernous sinus with the dimension of 11 × 10 × 10 mm (anteroposterior × right-left × craniocaudal); the residue showed T2 signal hyperintensity with no obvious change after radiotherapy (Figure 1A and 1B), there was no residual tumor in the sellar cavity and the left cavernous sinus was unremarkable. The pituitary stalk appeared centralized and the optic chiasm and sphenoid sinuses appeared unremarkable.

The patient had undergone an echocardiogram in 2018, which was normal. He had an ultrasound of his neck in January 2024, which showed normal slightly prominent thyroid gland with no definite nodule. He also underwent a colonoscopy in May 2024 that showed 5 colonic benign polyps that were resected. He did not have diabetes or hypertension.

## Treatment

The patient was initiated on octreotide LAR, 30 mg every 4 weeks because lanreotide was unavailable at our institute. On treatment initiation, his biochemical panel was: ACTH, 8.13 pmol/L (36.9 pg/mL) (normal range 1.59-13.94 pmol/L [7.2-63.3 pg/mL]; cortisol, 175.0 nmol/L (6.3 mcg/dL); 25 hydroxy vitamin D 15 nmol/L (6 ng/mL)(optimum value >50 nmol/L [20 ng/mL]; FSH, 5.6 IU/L (1.5-12.4 IU/L); LH, 3.6 IU/L (1.7-8.6 IU/L); prolactin, 4.32 μg/L (92 mIU/L); TSH, 1.16 mIU/L; free thyroxine-4, 10.7 pmol/L (8.31 ng/L); GH, 2.6 μg/L; IGF1, 58.96 nmol/L [451.0 μg/L]; and total testosterone, 6.62 nmol/L (191 ng/dL).

In February 2022 at the multidisciplinary team meeting to discuss his treatment, the surgical proposal was for a craniotomy followed by medical therapy; the radiation perspective was for watchful waiting for 5 years; and the endocrinological approach was to start on second-line treatment for acromegaly. The patient was counselled about the offered options and he decided to go for medical therapy. Given the lack of improvement with octreotide, the patient was switched to intramuscular pasireotide 40 mg IM every 4 weeks in August 2023.

## Outcome and Follow-up

**Figure 2.**
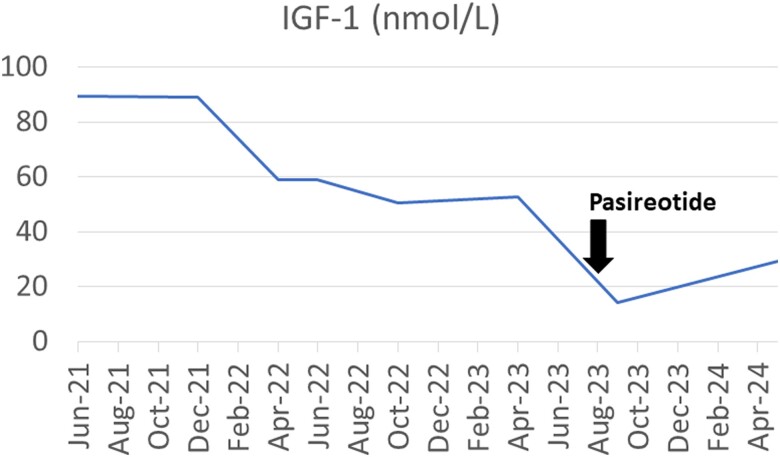
Changes in IGF1 over treatment course. Initiation of 40 mg pasireotide in August 2023.

One month after the first injection, his IGF1 level decreased to 14.12 nmol/L (108 μg/L)(Table 1, Fig. 2). The patient reported an immediate improvement in symptoms, with no subsequent headaches, the first time since his diagnosis 10 years previously. He reported no side effects, although his hemoglobin A1c increased from 5.6% to 5.9%.

**Table 1.** Biochemical panel over the course of treatment at our institute

Date	GH	IGF1
Normal range	0.4-10 μg/L (0.4–10 ng/mL)	12.94-31.25 nmol/L (99-239 μg/L)
June 1, 2021	6.67 μg/L (6.67 ng/mL)	H 89.42 nmol/L (H 684.0 μg/L)
December 15, 2021	5.99 μg/L (5.99 ng/mL)	H 89.16 nmol/L (H 682.0 μg/L)
April 28, 2022	2.60 μg/L (2.60 ng/mL)	H 58.96 nmol/L (H 451.0 μg/L)
June 26, 2022	2.91 μg/L (2.91 ng/mL)	H 58.96 nmol/L (H 451.0 μg/L)
October 10, 2022	1.24 μg/L (1.24 ng/mL)	H 50.46 nmol/L (H 386.0 μg/L)
April 16, 2023	—	52.69 nmol/L (H 403.0 μg/L)
September 3, 2023	3.71 μg/L (3.71 ng/mL)	14.12 nmol/L (108.0 μg/L)
May 6, 2024	6.65 ug/L (6.65 ng/mL)	29.15 nmol/L (223 µg/L)

Initiation of 40 mg pasireotide in August 2023.

Abbreviation: H, high.

**Figure 3.**
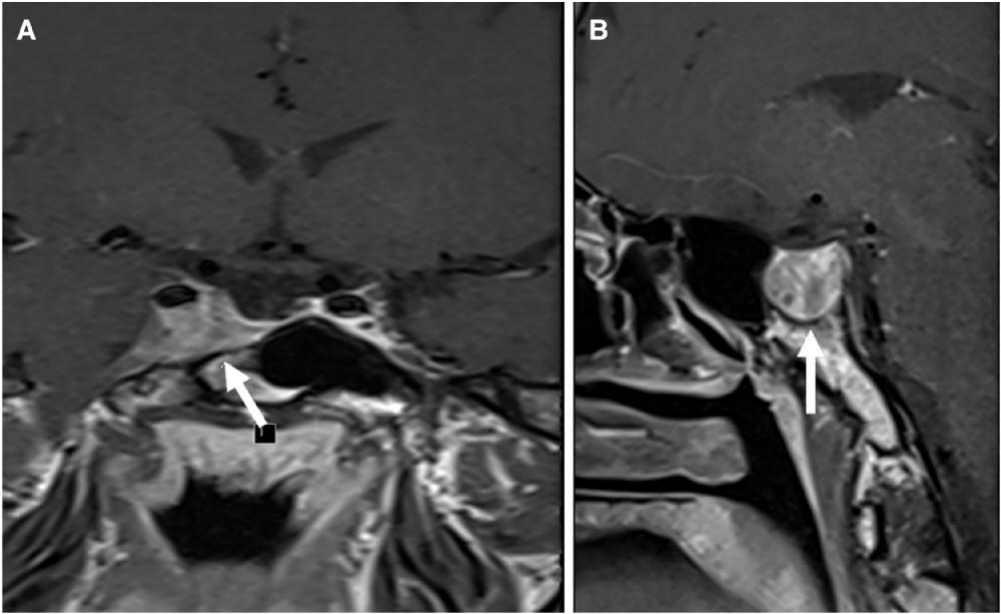
Cross-sectional (A) and sagittal (B) MRI scans showing residual pituitary tumor (December 2023).

Approximately 5 months after the start of treatment, the patient had a repeat MRI of the pituitary that showed redemonstration of irregular area of altered signal with contrast enhancement in the right cavernous sinus and surrounding the internal carotid artery. The residue measurement was unchanged at 11 × 10 × 10 mm but the T2 signal intensity looked brighter and there were more cystic changes indicating possible shrinkage (Figure 3).

His most recent follow-up was in May 2024. Clinically, he is still free from headache and reports no significant medication side effects.

## Discussion

This case report illustrates that persistent headache can be a significant burden to patients despite ongoing surgical, radiological, and medical treatment for pituitary adenoma.

GH-secreting pituitary tumors can be particularly pronociceptive (14). Treatment with somatostatin analogues is well established because of their analgesic effect beyond simply tumor size reduction, suggesting that somatostatin analogues have a biochemical inhibitory effect on pronociceptive peptides (14). In some cases, treatment with somatostatin analogues has resulted in headache alleviation but without biochemical control of acromegaly (14). In the present study, remarkably, within a month of switching medical treatment from octreotide to pasireotide, there was not only a notable reduction in IGF1 levels to 14.12 nmol/L (108 ug/L)(normal range, 12.94-31.25 nmol/L [99-239 µg/L]) but a complete resolution of headache, suggesting that for a certain patient subset, pasireotide can provide rapid biochemical and symptomatic improvement, in this case freeing the patient from this debilitating clinical manifestation of acromegaly and dramatically improving his quality of life.

Indeed, given its binding affinity for SST5, analgesic effects with pasireotide have long been postulated and were already identified in the phase II clinical study with pasireotide (15). In the 6-month study extension, the number of headache-free patients approximately doubled (16). These results suggested a marked biochemical control as well as improvement in headaches with pasireotide treatment. In the phase III, randomized comparative PAOLA study, comparing pasireotide with octreotide, similar improvements were reported in headache (mean, −0.3 [SD, 1.17] and −0.4 [SD, 0.94], respectively), and reports of headache as an adverse event were also lower in the pasireotide group than in the octreotide group (18.5% vs 25.6%) (2). In the PAOLA study extension, a switch from first-generation somatostatin analogues to pasireotide resulted in 22% of patients achieving disease control, whereas a pasireotide dose increase from 40 to 60 mg enabled 28% of patients to achieve disease control. All study groups had improvements in headache and other acromegaly-related symptoms (17). In the open-label PAPE study in 61 acromegaly patients, which showed that switching from first-generation somatostatin analogues to pasireotide monotherapy or a pasireotide and pegvisomant combination could control IGF1 levels in most patients, improvements in quality of life in a subset of patients on pasireotide were driven by improvements in fatigue and headache (18, 19). Moreover, in a real-life study of 35 acromegaly patients uncontrolled on first-generation somatostatin analogues and subsequently treated with pasireotide, 6 patients had severe headaches that resolved (n = 4) or significantly improved (n = 2) with pasireotide (20). In this study, irrespective of the hormonal response to pasireotide there was a significant decrease in headache frequency and intensity for all patients (20). The authors postulated that this effect may have been as a result of pasireotide's superior binding to SST5 rather than to SST2 (20). Finally, the efficacy of pasireotide in resolving acromegaly-related headache has been specifically studied in 3 case reports. Marina *et al*. reported on 2 Scandinavian cases, both of whom had severe headaches that were nonresponsive to octreotide who were switched to pasireotide and experienced rapid headache resolution, 1 with an accompanying improvement in biochemical results and 1 without (21). More recently, Lovato and Kapsner reported on 1 patient in New Mexico, USA, with intractable headache despite surgery, radiotherapy, and treatment with octreotide who had both biochemical and symptomatic resolution after switching to pasireotide (22).

Based on the preliminary evidence in acromegaly, experts have recommended using pasireotide in patients with headache not responsive to first-generation somatostatin receptor ligand therapy and in patients who experience side effects or are intolerant to pegvisomant monotherapy (23). The confirmation of pasireotide's rapid efficacy in the present case in both completely resolving headache and normalizing the patients’ IGF1 levels is confirmation of the previous findings. These results are particularly significant because there is a real, unmet clinical need and very real patient suffering. Up to 70% of patients do not achieve adequate biochemical control with first-generation somatostatin analogues (24). And for those patients who do achieve control on a first-generation somatostatin analogue, headache persisted in 71% of patients (14). There is thus clearly an ongoing need for better management of this subset of treatment-resistant acromegaly patients.

## Learning Points

- Acromegaly is often accompanied by frequent, debilitating headaches.
- In this case, despite surgery and treatment with first-generation somatostatin analogues and radiation therapy, severe frequent headaches persisted.
- Switching treatment to pasireotide achieved rapid and complete resolution of headaches and normalization of IGF1 levels within a month of the switch.
- Pasireotide has potential as a treatment option in acromegaly for patients with persistent headaches and inadequate response to other therapies.

## Data Availability

Original data generated and analyzed during this study are included in this published article.

## Abbreviations

MRI: magnetic resonance imaging
SSTR2: somatostatin receptor 2
SSTR5: somatostatin receptor 5
